# Molecular Determinants of Thyroid Cancer Progression: Thyroid Hormone Signaling, the BRAF/MAPK Pathway, and Emerging miRNA Biomarkers

**DOI:** 10.3390/biomedicines14050967

**Published:** 2026-04-23

**Authors:** Marina Lasa, Constanza Contreras-Jurado

**Affiliations:** 1 Instituto de Investigaciones Biomédicas Sols-Morreale (IIBM), Consejo Superior de Investigaciones Científicas—Universidad Autónoma de Madrid (CSIC_UAM), 28029 Madrid, Spain; mlasa@iib.uam.es; 2 Departamento de Bioquímica, Facultad de Medicina, Universidad Autónoma de Madrid, 28029 Madrid, Spain; 3 Facultad de Ciencias Biomédicas y de la Salud, Universidad Alfonso X El Sabio, Villanueva de la Cañada, 28691 Madrid, Spain; 4 Centro de Investigación Biomédica en Red de Cáncer (CIBERONC), Instituto de Salud Carlos III, 28029 Madrid, Spain

**Keywords:** thyroid cancer, thyroid hormones, MAPK, ^V600E^BRAF, PI3K/AKT, miRNAs

## Abstract

Thyroid cancer is the most common malignancy of the endocrine system and represents a biologically heterogeneous disease driven by the interplay between endocrine regulation, oncogenic signaling pathways, and tumor microenvironment dynamics. Although most follicular cell-derived thyroid cancers follow an indolent clinical course, a subset progresses toward aggressive, therapy-refractory phenotypes, underscoring the need for refined molecular understanding and improved biomarkers. This review comprehensively examines the molecular determinants of thyroid cancer progression, with particular emphasis on Thyroid Hormone (TH) signaling, the Mitogen-Activated Protein Kinase (MAPK) and Phosphoinositide 3-Kinase (PI3K)/AKT pathways, and the emerging role of microRNAs (miRNAs). We discuss how oncogenic alterations, most notably the ^V600E^BRAF mutation, act as central drivers of tumor initiation and aggressiveness by sustaining MAPK/ERK signaling, promoting dedifferentiation, metabolic reprogramming, immune evasion, and resistance to targeted therapies. The cooperative role of PI3K/AKT signaling in reinforcing survival, invasion, and treatment resistance is highlighted, emphasizing the network-level integration of oncogenic pathways rather than linear dependency on single drivers. In parallel, thyroid hormones exert context-dependent effects on tumor biology through both genomic actions mediated by nuclear thyroid hormone receptors and non-genomic mechanisms initiated at the integrin αvβ3 receptor, linking endocrine status to cancer progression and therapeutic response. Finally, we review the expanding evidence supporting miRNAs as critical regulators of thyroid carcinogenesis and as promising diagnostic, prognostic, and predictive biomarkers. The clinical validation of miRNA-based panels and circulating miRNAs offers new opportunities to improve preoperative risk stratification, reduce overtreatment, and guide personalized therapeutic strategies. Collectively, these insights support a multidimensional framework for understanding thyroid cancer progression and highlight future directions for precision oncology.

## 1. Introduction

### 1.1. Thyroid Gland and Thyroid Hormones

**Figure 1:**
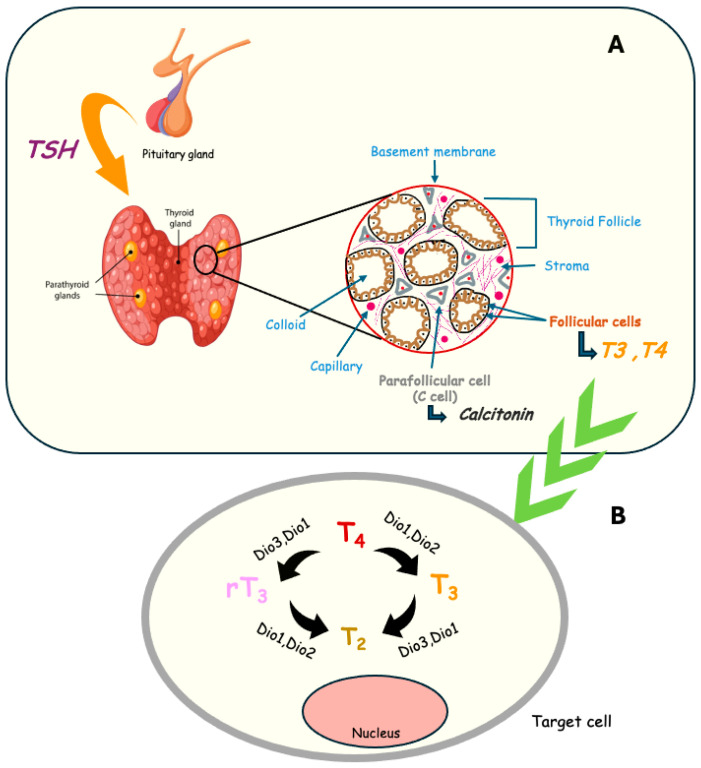
(**A**) Thyroid gland composition. The structural and functional unit of the thyroid gland is the thyroid follicle, which is composed of follicular cells. TSH acts on thyroid follicular cells, promoting the production and release of the THs, T4 and T3. (**B**) Local conversion of T4 into T3. The conversion of T4 of most biologically active hormone T3 in target tissues is facilitated by deiodinases.

The thyroid gland is an endocrine organ located in the anterior neck below the larynx, composed of two lateral lobes connected by an H-shaped isthmus, giving it a butterfly-like appearance. Each lobe consists of numerous thyroid follicles, the structural and functional units of the gland, formed by follicular cells that secrete THs and store a substance called colloid containing thyroglobulin. Between the thyroid follicles are the parafollicular cells, or C cells, which secrete the polypeptide hormone calcitonin, whose main function is the regulation of calcium and phosphate levels in the blood and skeletal tissue. This gland is highly vascularized, as the hormones it secretes must exert their action on target cells distributed throughout the body [1] (Figure 1A).

Thyroid function is regulated by the hypothalamic–pituitary–thyroid axis, in which the hypothalamus releases Thyroid-Releasing Hormone (TRH) to stimulate pituitary secretion of Thyroid-Stimulating Hormone (TSH), with both hormones controlled by negative feedback from circulating THs. TSH promotes synthesis and release of Thyroxine (T4) and Triiodothyronine (T3) in thyroid follicular cells, a process that requires dietary iodine absorbed in the intestine and concentrated in the thyroid. Inside the thyroid, iodide undergoes oxidation in a reaction catalyzed by Thyroid Peroxidase (TPO) in the presence of hydrogen peroxide. The activated iodine is then attached to specific tyrosine residues within thyroglobulin, generating Monoiodotyrosine (MIT) and Diiodotyrosine (DIT). Subsequent coupling reactions between these iodinated residues lead to the formation of the THs T3 and T4. T3 represents the most biologically active form of THs, while T4 exhibits significantly lower biological activity. THs are secreted through the basolateral membrane by the active transmembrane MonoCarboxylate Transporter 8 (MCT8). Peripheral tissue availability of active T3 depends largely on local conversion of T4 by selenium-containing Deiodinase enzymes (DIO) enzymes, which regulate TH activation and inactivation [2]. DIO1 and DIO2 convert T4 to T3, whereas DIO3 inactivates THs by producing Reverse T3 (rT3) and Diiodo-L-thyronine (T2), maintaining a balance between active and inactive hormone forms [3]. Through these complementary actions, DIOs maintain the balance between active and inactive TH forms (Figure 1B).

THs play essential roles in growth, development, and metabolic regulation, and most of their biological effects are mediated through interactions with nuclear TRs, which function as ligand-dependent transcription factors [4,5,6]. TRs belong to the nuclear receptor superfamily and are encoded by two genes, *TRA* and *TRB*, which generate multiple receptor isoforms through alternative promoters and splicing [7,8]. The *TRA* gene produces TRα1, a functional hormone-binding receptor, and TRα2, which lacks high-affinity hormone binding and may modulate receptor signaling. Additional truncated variants, some located outside the nucleus, suggest potential non-genomic roles. The *TRB* gene mainly encodes the hormone-binding isoforms TRβ1 and TRβ2, along with other minor variants whose functions are not yet fully understood. The principal functional isoforms—TRα1, TRβ1, and TRβ2—exhibit distinct tissue-specific and developmental expression patterns that underlie differential THs responses [9,10,11]. TRα1 is predominantly expressed in heart, brain, and skeletal muscle and is essential for postnatal growth and cardiac function. TRβ1 is widely expressed, particularly in liver, kidney, and pituitary, where it regulates metabolism and feedback control of the hypothalamic–pituitary–thyroid axis. TRβ2 shows restricted expression, mainly in the anterior pituitary and certain neuronal populations, where it contributes to sensory organ development.

Activated TRs regulate gene expression by recruiting coregulatory proteins, enabling both transcriptional activation and repression. Beyond these genomic actions, THs also elicit rapid non-genomic responses mediated by extra-nuclear TRs or plasma membrane–associated receptors such as integrin αvβ3. Strong evidence links non-genomic TR actions to activation of PI3K and its downstream PI3K/Akt signaling cascade, which controls multiple cellular processes [12]. Additional signaling pathways involved in TR action include MAPK- and Dual Specificity Phosphatase 1 (DUSP1)-mediated pathways [13,14] and Nuclear Factor Kappa B (NF-κB) signaling [14,15,16].

### 1.2. Thyroid Cancer

Thyroid cancer is the most common malignant tumor of the endocrine system, and its incidence has increased markedly over recent decades worldwide. Thyroid malignancies can arise from different cellular components of the gland, therefore, there are currently eight categories for classifying tumors organized according to the cell of origin, pathological characteristics (cytology and histology), and molecular profiles [17]:

- Developmental abnormalities
- Follicular cell-derived neoplasms
- Thyroid C-cell-derived carcinoma
- Mixed medullary and follicular cell-derived carcinomas
- Salivary gland-type carcinomas of the thyroid
- Thyroid tumors of uncertain histogenesis
- Thymic tumors within the thyroid
- Embryonal thyroid neoplasms

Thyroid tumors derived from thyroid follicular cells are the most frequent. In the last edition (5th 2022) of the World Health Organization (WHO) Classification of Endocrine and Neuroendocrine Tumors, tumors derived from thyroid follicular cells have undergone in a new classification based on three broad prognostic categories: benign, low-risk, and malignant neoplasms [18] (Table 1).

*Benign neoplasms* originating from thyroid follicular cells comprise various nodular and adenomatous lesions. Although multinodular enlargement of the thyroid has historically been interpreted as a hyperplastic process, molecular findings indicate that many of these nodules are monoclonal, consistent with true benign tumors. To better encompass this biological overlap, the most recent WHO classification adopts the term FND. Within the benign category, recognized entities include classic follicular adenoma and follicular adenoma with papillary growth patterns. The latter represents a well-defined, noninvasive tumor lacking the characteristic nuclear features of papillary thyroid carcinoma and is frequently associated with alterations that activate the protein kinase A signaling pathway. Oncocytic follicular adenomas display distinctive molecular features—particularly mitochondrial DNA abnormalities and chromosomal copy number changes—supporting their classification as a distinct subgroup of thyroid tumors.

*Low-risk follicular cell-derived thyroid neoplasms* was established to describe a subset of tumors whose morphological and molecular characteristics are consistent with indolent clinical behavior. In the 5th edition of the WHO classification, this group has been further defined and broadened to include noninvasive NIFTP, FT-UMP and WD-UMP, and HTTs. When diagnosed according to stringent histopathological criteria, these lesions typically follow a favorable course and do not warrant aggressive therapeutic approaches.

*Malignant thyroid neoplasms* include PTC, FTC, IEFVPTC, HGDTC, OTC, PDTC, ATC. In FTC-related tumors, the extent of capsular and vascular invasion remains a key determinant of clinical outcome. A significant update is the recognition of high-grade carcinomas, defined by elevated mitotic activity and/or tumor necrosis, which biologically fall between well-differentiated carcinomas and ATC. The grading approach has also been extended to medullary thyroid carcinoma, where proliferative markers and necrosis help predict prognosis are central to tumor development [18,19,20,21].

Treatment of thyroid tumors is variable depending on the type, stage, and individual patient factors. The most common treatment is surgery, which may involve removing either part of the thyroid or the entire gland. Surgical resection remains the primary modality for well-differentiated thyroid cancers and is associated with excellent long-term survival, though extent of surgery should be tailored to tumor characteristics and risk factors [22]. After surgery, patients usually require lifelong TH replacement therapy to regulate metabolism and suppress TSH, which may reduce the risk of recurrence. Hormone suppression therapy is a standard part of post-operative management to optimize metabolic regulation and mitigate recurrence risk. (standard endocrinology practice) TSH suppression is widely recommended in clinical guidelines. Another common treatment is Radioactive Iodine (RAI) therapy, which is used after surgery to destroy residual thyroid cancer tissue, although it is not useful for more aggressive forms, such as ATC or medullary thyroid carcinoma, which are typically RAI-refractory [23]. In more advanced or resistant cases, systemic therapies, including targeted drugs, may be used. Tyrosine kinase inhibitors have been shown to significantly prolong progression-free survival in patients with RAI-refractory differentiated thyroid cancer compared with placebo, indicating a role in advanced disease. However, although these therapies can improve progression-free survival, they often come with significant adverse events [24]. Despite the availability of multiple treatments, there are important limitations: (1) recurrence is common even after apparently successful initial therapy, with long-term follow-up showing disease return in a proportion of patients with differentiated thyroid carcinoma [22]; (2) aggressive subtypes such as PDTC or ATC grow rapidly and are not responsive to standard RAI therapy, which limits therapeutic options; such patients often require alternative systemic or palliative approaches [23]; and (3): long-term hormone therapy requires careful monitoring to avoid complications. For these reasons, while the prognosis for many thyroid cancer patients is favorable, ongoing research is essential to develop more effective and less toxic treatment options.

### 1.3. Molecular Mechanisms and Signaling Pathways Involved in Thyroid Cancer

The “hallmarks of cancer” framework defines the essential biological capabilities acquired during tumorigenesis and has become a central model for understanding cancer across tumor types [25]. These hallmarks include sustained proliferative signaling, resistance to growth suppression and cell death, angiogenesis, invasion and metastasis, metabolic reprogramming, and immune evasion, with later updates highlighting genome instability, tumor-promoting inflammation, phenotypic plasticity, and the influence of the tumor microenvironment [26]. Thyroid cancer exhibits several of these hallmarks at the molecular level, with PTC showing proliferative signaling, apoptosis resistance, and genomic and epigenetic alterations, while advanced and ATC display enhanced angiogenesis and immune evasion. Metabolic reprogramming is particularly evident in PDTC, and these features have been linked to potential targeted therapies [27]. Among the signaling pathways driving these hallmarks, MAPK/ERK and PI3K/AKT are especially important in thyroid cancer. The MAPK/ERK pathway is typically activated by growth factor binding to Tyrosine Kinase Receptors (RTKs), leading to sequential activation of RAS, RAF (particularly BRAF), MEK, and ERK, ultimately regulating gene expression involved in proliferation and survival [28,29,30,31,32,33]. Oncogenic mutations such as ^V600E^BRAF cause constitutive ERK signaling independent of external stimuli and are central to thyroid tumorigenesis [34]. The PI3K/AKT pathway, another key regulator of cell growth, survival, metabolism, and proliferation, is frequently dysregulated in thyroid cancers, especially FTC, PDTC, and ATC [34,35,36,37]. Its activation through RTKs leads to PI3K-dependent generation of Phosphatidylinositol-3,4,5-trisphosphate (PIP3) and subsequent AKT activation, promoting apoptosis inhibition, cell cycle progression, and metabolic changes that contribute to thyroid cancer development and progression [35,36].

## 2. Oncogenic Signaling Networks in Thyroid Cancer: MAPK/ERK and PI3K/AKT Pathways

Mutations in the RAS family are among the most frequent genetic alterations in thyroid cancer, particularly in FTCs, where they contribute to tumor initiation and progression. Codon 61 mutations in *NRAS* are especially relevant, as they impair intrinsic GTPase activity and lock RAS in a constitutively active state, sustaining proliferative signaling through both MAPK and PI3K/AKT pathways [38]. In addition, activating mutations in *BRAF*—most notably V600E—represent the dominant oncogenic event in PTCs and ATCs [39]. This mutation mimics activation loop phosphorylation, stabilizing BRAF in a constitutively active conformation and driving persistent ERK/MAPK signaling. As a result, ^V600E^BRAF not only promotes uncontrolled proliferation but also induces dedifferentiation and enhances metastatic potential [39,40]. Clinically, this mutation is strongly associated with poor prognosis and recurrence, underscoring its role as both a biomarker and therapeutic target [41]. Beyond MAPK signaling, the PI3K/AKT pathway cooperates with BRAF-driven signaling to amplify oncogenic outputs, including survival, proliferation, metabolic adaptation, and therapy resistance [42]. Its activation arises through multiple mechanisms—mutant RAS, PIK3CA gain-of-function mutations, or Phosphatase and Tensin homolog deleted on chromosome ten (PTEN) loss—and frequently coexists with MAPK dysregulation, generating synergistic signaling [42]. Importantly, in BRAF-mutant tumors, concurrent PI3K/AKT alterations are associated with more aggressive disease and worse outcomes, highlighting that tumor behavior is dictated by integrated network activity rather than single mutations [43].

### 2.1. Contribution to Cell Invasion and Metastasis

^V600E^BRAF is a central driver of thyroid cancer invasion through sustained ERK activation, which promotes Epithelial–Mesenchymal Transition (EMT) via transcription factors such as Snail, leading to E-cadherin repression and loss of cell adhesion [44]. This MAPK-driven program is reinforced by PI3K/AKT signaling, which enhances cytoskeletal remodeling, motility, and resistance to apoptosis [34]. Mechanistically, PI3K activation induces PIP3 accumulation and AKT phosphorylation, regulating effectors such as Glycogen Synthase Kinase-3 Beta (GSK3β) and mammalian Target of Rapamycin (mTOR) that control actin dynamics and focal adhesion turnover [34]. Additional alterations, including PTEN loss or PIK3CA mutations, further amplify this invasive phenotype [42]. Transforming Growth Factor-β (TGF-β) signaling synergizes with BRAF-driven MAPK activity by activating MAPK and stored response chain (Src)/Focal Adhesion Kinase (FAK) pathways and stabilizing EMT programs [45,46]. Moreover, ^V600E^BRAF engages inflammatory mediators such as NF-κB, promoting extracellular matrix remodeling and pro-invasive signaling [47], an effect further strengthened by PI3K/AKT-mediated NF-κB activation [48].

Clinically, ^V600E^BRAF correlates with lymph node metastasis and aggressive behavior in PTC, although its independent prognostic value remains under investigation [49,50,51]. Notably, co-activation of PI3K/AKT is consistently linked to advanced disease and reduced therapeutic response, emphasizing the importance of MAPK–PI3K crosstalk [34,37].

### 2.2. Role in Tumor Microenvironment and Immune Evasion

^V600E^BRAF acts as a dominant regulator of Tumor Microenvironment (TME) remodeling by driving constitutive MAPK signaling and orchestrating tumor–stroma interactions. Through transcriptional reprogramming, it promotes a secretory and invasive phenotype that enhances extracellular matrix remodeling and stromal activation [52,53]. Cancer-Associated Fibroblasts (CAFs) further reinforce this process by secreting cytokines and matrix-remodeling factors, establishing a reciprocal loop that amplifies both MAPK and PI3K/AKT signaling. Within this framework, PI3K/AKT primarily functions as a cooperative pathway that supports survival, stress adaptation, and cytokine production rather than initiating TME remodeling. Its activation enhances tumor fitness under microenvironmental stress and reinforces MAPK-driven phenotypes [52]. Importantly, ^V600E^BRAF-driven ERK activity is a key determinant of immune evasion through Programmed Death-Ligand 1(PD-L1) upregulation, promoting T cell exhaustion [54,55]. PI3K/AKT further supports this immunosuppressive state by enhancing checkpoint expression and survival signaling [56]. In parallel, cytokines such as Interleukin-6 (IL-6), Interleukin-10 (IL-10), and TGF-β recruit immunosuppressive populations, including Myeloid-Derived Suppressor Cells (MDSCs) and Regulatory T cells (Tregs), while M2 macrophages promote angiogenesis and invasion [57]. Notably, ^V600E^BRAF is associated with increased chemokine expression (e.g., C-C motif ligand 2 (CCL2)), enhancing macrophage recruitment and reinforcing a pro-tumorigenic niche [58]. Overall, ^V600E^BRAF emerges as the central coordinator of tumor plasticity, TME remodeling, and immune escape, with PI3K/AKT acting as a reinforcing axis.

### 2.3. Effects on Metabolic Reprogramming and Autophagy

^V600E^BRAF is a master regulator of metabolic reprogramming in thyroid cancer, promoting glycolysis, mitochondrial adaptation, lipogenesis, and glutamine dependence [34,37]. Through constitutive MAPK signaling, it upregulates Glucose Transporter type 1(GLUT1), glycolytic enzymes, and Hypoxia-Inducible Factor 1-Alpha (HIF-1α) activity, reinforcing the Warburg effect [59,60,61]. PI3K/AKT signaling complements this program by enhancing glucose uptake, glycolytic flux, and anabolic metabolism, while converging on mTOR to integrate nutrient sensing with biosynthesis [62,63]. Together, these pathways sustain tumor growth and redox balance. Despite partial reduction in Mitochondrial Oxidative Phosphorylation (OXPHOS), mitochondrial function is preserved to support biosynthesis [64]. Autophagy further maintains metabolic flexibility and mediates resistance to BRAF inhibition, often induced via AMPK-dependent mechanisms [65,66,67]. PI3K/AKT/mTOR signaling tightly regulates this process, suppressing autophagy under nutrient-rich conditions but allowing compensatory activation under stress [68]. ^V600E^BRAF also promotes lipid synthesis and glutamine metabolism, with PI3K/AKT reinforcing these anabolic pathways [63,69,70]. This metabolic rewiring contributes to immune evasion, as increased glycolysis leads to lactate accumulation, TME acidification, and impaired T-cell function [71].

Thus, ^V600E^BRAF drives metabolic reprogramming, while PI3K/AKT stabilizes and amplifies these adaptations, supporting survival and immune escape.

### 2.4. Reassessing the Oncogenic and Therapeutic Centrality of ^V600E^BRAF in Thyroid Cancer

Although ^V600E^BRAF is a key tumor-initiating driver, its role in tumor maintenance is increasingly questioned. Clinical responses to BRAF inhibitors in thyroid cancer are often partial and transient, reflecting adaptive resistance mechanisms [72,73]. A major limitation of the BRAF-centric model is the assumption of linear MAPK dependency. In reality, ERK signaling is dynamically maintained through network-level feedback. BRAF inhibition relieves ERK-dependent negative feedback, leading to RTK and RAS reactivation and rapid restoration of MAPK signaling [74]. Additional mechanisms—RAF dimerization, isoform switching, and secondary mutations—further sustain ERK activity independently of BRAF [75].

In parallel, activation of alternative pathways such as PI3K/AKT/mTOR redistributes signaling flux and contributes to resistance [73,76]. Tumor cells also exhibit phenotypic plasticity, undergoing dedifferentiation and transcriptional reprogramming toward drug-tolerant states less dependent on MAPK signaling [76].

Moreover, metabolic adaptation and autophagy provide parallel survival routes that further reduce dependence on BRAF signaling [77]. Even downstream inhibition of MEK or ERK fails to achieve durable suppression due to pathway resilience and non-canonical signaling [78].

## 3. Thyroid Hormones and Thyroid Cancer

THs are key regulators of physiological processes, including cellular growth, energy metabolism, differentiation, and programmed cell death. Increasing evidence over the past decades indicates that their influence extends beyond normal physiology to the modulation of cancer development and progression. Experimental studies have shown that both T3 and T4 can enhance tumor cell proliferation, invasiveness, angiogenesis, and survival in multiple malignancies. These actions are mediated not only by traditional nuclear TRs but also by rapid, non-genomic pathways initiated at the cell membrane through integrin αvβ3, which is highly expressed in malignant cells and actively proliferating endothelial cells. In addition, the local hormonal milieu within the tumor microenvironment is biologically relevant. Altered systemic thyroid states, such as hyperthyroidism and hypothyroidism, have been associated with differences in cancer risk and clinical outcomes. Epidemiological data frequently associate hyperthyroidism with an elevated incidence of several solid tumors, whereas hypothyroidism has been linked in some contexts to delayed tumor development or reduced aggressiveness [79].

Multiple studies—including cohort investigations [79,80], meta-analyses [81,82,83], and case–control analyses [84,85]—have highlighted the association between TSH, T4 (total and free), and T3 (total and free) levels and the development of thyroid cancer.

### 3.1. Thyroid Hormones

THs exert a dual role in cancer through distinct molecular pathways with opposing effects. Although T4 is traditionally viewed only as a prohormone for T3, at the cell surface it acts as the primary ligand for a receptor located on the extracellular domain of integrin αvβ3. The non-genomic pathway is initiated when T3 or T4 bind to the integrin αvβ3 receptor on the cell membrane, leading to activation of MAPK/ERK and PI3K/Akt signaling. Structurally, this receptor contains two distinct binding domains: S1, which specifically binds T3 to activate the PI3K/Akt pathway, and S2, which primarily binds T4 (and T3 with lower affinity) to activate the MAPK/ERK cascade [85,86]. This promotes cell proliferation, angiogenesis, metastasis, apoptosis resistance, and immune evasion, while upregulating proteins such as Cyclin D1 (CD1), Vascular Endothelial Growth Factor (VEGF), Matrix Metalloproteinases (MMPs), and PD-L1 [86,87,88,89,90]. T3 is the active form of the hormone for genomic signaling, but its role in thyroid cancer is complex and can be dualistic. At physiological levels, T3 acts through nuclear receptors (TRα and TRβ) to promote the expression of differentiation-related genes and can exert an anti-proliferative effect on tumor growth [79,86]. However, *TRβ* often acts as a tumor suppressor that is silenced or mutated in many cancers [89]. A notable example is the dominant-negative *PV* mutation, which disrupts T3 binding and allows the receptor to interact anally with the p85α subunit of PI3K, constitutively activating oncogenic signaling and promoting growth [86].

Unlike T4, T3 has little bioactivity at the integrin αvβ3 receptor at physiological concentrations. T3 can promote aerobic glycolysis, allowing cancer cells to satisfy energetic demands even in the presence of oxygen, a hallmark of cancer progression known as the Warburg effect [79,91]. In advanced cases of differentiated thyroid carcinoma, higher T3 levels have been negatively correlated with survival and associated with a poorer prognosis [85].

T4 has direct pro-tumorigenic role in thyroid cancer through non-genomic mechanisms [79,90]. It is the principal ligand for a hormone receptor located on the extracellular domain of integrin αvβ3 a protein that is overexpressed and activated in cancer cells and rapidly dividing endothelial cells. T4 binding to this receptor stimulates the proliferation of papillary and follicular thyroid cancer cells. It also activates anti-apoptotic defense pathways by blocking the accumulation of pro-apoptotic proteins like p21, c-Fos, and c-Jun. T4 promotes the formation of new blood vessels (angiogenesis) by upregulating factors such as VEGF-A and basic Fibroblast Growth Factor (bFGF). Furthermore, T4 can induce radio-resistance through conformational changes in the integrin receptor. T4 supports the metastatic process by enhancing cell migration and fostering interactions between platelets and tumor cells [86,90].

### 3.2. Thyroid-Stimulating Hormone

TSH is a well-recognized growth regulator of thyroid follicular cells and is considered a primary growth factor for Differentiated Thyroid Cancers (DTC), particularly papillary and follicular thyroid carcinoma. TSH exerts its biological effects by binding to the Thyroid-Stimulating Hormone Receptor (TSHR), a specific G protein–coupled receptor located on the surface of thyroid and tumor cells. Activation of this receptor initiates intracellular signaling cascades, including the cAMP/PKA and PI3K/AKT pathways, which promote cell proliferation and stimulate the synthesis of thyroid-specific proteins [86,90]. Consistent with this mechanism, elevated serum TSH levels have been significantly associated with an increased risk of developing differentiated thyroid cancer and are often correlated with more advanced tumor stages at the time of diagnosis [81,92]. Quantitative analyses further indicate that progressive increases in circulating TSH are accompanied by a proportional rise in malignancy risk, with supraphysiological concentrations exerting the strongest effect. However, the clinical significance of TSH as a risk factor is modified by the presence of Autoimmune Thyroiditis (AT), also known as Hashimoto’s thyroiditis. AT is the leading cause of acquired hypothyroidism and is characterized histologically by lymphoplasmacytic infiltration and clinically by the presence of thyroid autoantibodies, specifically Thyroid Peroxidase Antibody (TPOAb) and Thyroglobulin Antibody (TgAb). In euthyroid individuals without AT or positive autoantibodies, there is a significant positive association between higher serum TSH levels and an increased risk of DTC. In stark contrast, this association between TSH levels and cancer risk disappears in patients with concurrent AT. This discrepancy suggests that in the context of autoimmunity, inflammatory and immune-related pathways may drive thyroid tumor development through mechanisms independent of classical TSH signaling. Interestingly, DTC coexisting with AT often presents a less aggressive clinical profile and demonstrates improved prognostic outcomes compared to cases without autoimmunity [92].

Interestingly, some studies have also reported an association between suppressed TSH levels and increased thyroid cancer incidence in certain prospective populations, particularly among men. This apparent paradox suggests that subclinical hyperthyroid states may also contribute to tumor development. Within the physiological reference range, relatively lower TSH concentrations have been linked to higher cancer occurrence, whereas higher-normal values may exert a protective effect. Moreover, sex-specific patterns appear to influence this relationship, with low TSH levels being more strongly associated with cancer risk in women, while elevated TSH may represent a more relevant risk factor in men [80,92,93,94].

Given the central role of TSH in stimulating thyroid cell proliferation, standard clinical management for differentiated thyroid cancer commonly includes TSH suppression therapy. This strategy involves the administration of supraphysiological doses of levothyroxine (T4) to reduce circulating TSH levels and thereby minimize the proliferative stimulus on residual or recurrent tumor cells [86,92]. A critical balance exists between TSH-dependence and T4-dependence in thyroid tumors. For most patients, suppressing TSH with T4 successfully arrests tumor growth. However, if a thyroid cancer continues to grow despite full TSH suppression, the tumor may have shifted to becoming T4-dependent, utilizing the integrin αvβ3 pathway to drive its progression [89,90].

## 4. miRNAs in Thyroid Cancer

Most thyroid malignancies are well-differentiated and associated with favorable outcomes; however, a minority of cases, particularly ATC, exhibit aggressive behavior and poor survival. In recent years, the diagnostic landscape has evolved considerably, driven by progress in high-resolution ultrasonography, Fine-Needle Aspiration Cytology (FNAC), structured risk assessment systems such as Thyroid Imaging Reporting and Data System (TIRADS), and the incorporation of molecular and serum biomarkers. Today, the evaluation of thyroid nodules involves an integrated approach that combines clinical examination, imaging studies, laboratory tests, cytopathological assessment, and molecular profiling. Ultrasound serves as the first-line imaging tool, whereas fine-needle aspiration biopsy is central to distinguishing benign from malignant lesions. Molecular alterations identified through next-generation sequencing, along with markers such as thyroglobulin and calcitonin, contribute to disease monitoring and tailored therapeutic planning. Nevertheless, important issues persist, particularly the risk of overdiagnosis and overtreatment, highlighting the need for more accurate risk stratification to guide appropriate clinical management [95]. miRNAs represent a promising non-invasive biomarker for the diagnosis, prognosis, and monitoring of thyroid cancer since they are highly stable and can be identified in cytological and histological specimens, even when only minimal quantities of fresh or archived samples are available, as well as circulating blood simples [96]. miRNA dysregulation plays a major role in thyroid carcinogenesis. In thyroid neoplasms, approximately 32% of identified human miRNAs show increased expression, while about 38% are reduced by more than twofold relative to normal thyroid tissue [97]. Additionally, miRNA expression profiles vary markedly across thyroid cancer subtypes, even when tumors arise from the same cellular origin, highlighting their potential utility in tumor classification and biological characterization.

### 4.1. Oncogenic and Tumor Suppressors microRNAs

miRNAs act either as oncogenes (oncomiRs) or tumor suppressors. *OncomiRs*, or oncogenic microRNAs, are a subset of miRNAs that promote tumorigenesis by targeting tumor suppressor genes or activating oncogenic signaling pathways.

In thyroid cancer, oncomiRs contribute to uncontrolled proliferation, invasion, dedifferentiation, and metastasis through modulation of key pathways such as PI3K/AKT/mTOR, MAPK/ERK, β-catenin, and NF-κB signaling [98,99,100]. Among the most extensively studied oncomiRs, miR-146b is one of the most consistently overexpressed miRNAs in PTC. It promotes cell migration and invasion by directly targeting SMAD4, leading to dysregulation of the TGF-β signaling pathway. High miR-146b expression has been significantly associated with aggressive clinicopathological features, including extrathyroidal extension and the presence of the ^V600E^BRAF mutation Its overexpression inhibits the tumor suppressor PTEN, leading to hyperactivation of the PI3K/AKT signaling pathway, which is essential for cell survival and uncontrolled cellular proliferation [98,101].miR-21, miR23a, and miR-181a also inhibit PTEN [98,100]. miR-221 and miR-222, two homologous microRNAs, are frequently upregulated in both PTC and ATC. They drive tumor progression by targeting key regulators of the cell cycle, including Cyclin-Dependent Kinase Inhibitor Subtype 1B (CDKN1B) and the proto-oncogene KIT, thereby facilitating accelerated entry into the S phase. Clinically, their overexpression correlates with lymph node metastasis and increased risk of recurrence. miR-155 acts as an oncomiR by targeting the tumor suppressor APC, resulting in activation of the β-catenin pathway and downstream oncogenic effectors such as c-MYC. Downregulates TP53INP1, which promotes EMT. In ATC, increased miR-155 expression is strongly associated with extrathyroidal invasion [98,99,101]. The miR-17–92 cluster, particularly miR-19a, is overexpressed in aggressive thyroid cancers. This cluster promotes tumorigenesis by targeting PTEN, leading to activation of the PI3K/AKT/mTOR signaling pathway and enhanced cell survival and growth value [98,100,101]. In papillary thyroid carcinoma, miR-34a acts as an oncomiR by targeting GAS1 to activate the PI3K/Akt/Bad survival pathway [99]. miR-625-3p functions as an oncogenic microRNA in thyroid cancer by upregulating Astrocyte Elevated Gene 1 (AEG-1), which activates the WNT and JNK signaling pathways, enhances tumor cell migration, invasion, and proliferation, promotes drug tolerance, and suppresses apoptosis, as evidenced by the inverse regulation of pro-apoptotic factors such as BAX, CASP3, and CASP9.

Additional oncomiRs, including miR-21, miR-181a, and miR-223, have been associated with increased tumor size, lymph node metastasis, higher recurrence risk, and specific histological variants, supporting their potential diagnostic and prognostic value [101].

*Tumor suppressor* miRNAs, in contrast, are frequently downregulated in thyroid cancer. Their loss enhances malignant behavior. These miRNAs normally inhibit oncogenic pathways such as MAPK, PI3K/AKT, NF-κB, and β-catenin signaling. The Let-7 family is commonly downregulated in thyroid cancer and exerts tumor-suppressive effects by targeting the RAS oncogene, thereby inhibiting MAPK signaling, reducing cell proliferation, and promoting differentiation [98,99]. miR-9, miR-675, and miR-4728 also block MAPK signaling pathway by inhibiting BRAF, MAPK1, and SOS1 respectively. miR-15a is traditionally classified as a tumor suppressor microRNA that targets the anti-apoptotic protein BCL-2. By reducing BCL-2 expression, miR-15a promotes apoptosis and regulates cell proliferation through the AKT pathway. However, its role in thyroid cancer remains controversial, as context-dependent and sometimes opposing effects have been reported depending on tumor subtype and molecular background [98,101]. miR-497, miR-29a, miR-145, miR-217, miR-203, and miR-338-3p are involved in the regulation of thyroid cancer progression by targeting AKT3, a key component of the PI3K/AKT signaling pathway. They are frequently downregulated in thyroid cancer, and their restoration has been shown to significantly inhibit cell migration and invasion through the direct suppression of AKT3 [98,100]. miR-206 is frequently downregulated in thyroid cancer, particularly in aggressive subtypes. Restoration of miR-206 expression suppresses migration and invasion of PTC and ATC cells by targeting genes such as Myocardin Related Transcription Factor A (MRTFA) and Ras-related protein Rap-1b (RAP1B) [98]. miR-128 functions as a tumor suppressor by targeting Sphingosine Kinase-1 (SPHK1). Experimental overexpression of miR-128 has been shown to reduce tumor growth and tumor weight in thyroid cancer animal models [98,101]. miR-26a is often lost in anaplastic thyroid carcinoma, and its reintroduction inhibits tumor growth by downregulating Enhancer of Zeste Homolog 2 enzyme (EZH2), a key epigenetic regulator involved in transcriptional silencing during neoplastic progression [98]. miR-200 family and miR-214 act as a tumor suppressor in thyroid cancer inhibiting EMT and suppress migration and invasion. miR-200 exerts its effects by targeting ZEB1 and ZEB2, whereas miR-214 acts on PSMD10 within the proteasome pathway [98,100,102].

Other tumor suppressor miRNAs, including miR-375, miR-137, miR-153-3p, have also been reported to inhibit thyroid tumor progression through diverse oncogenic pathways, although their precise contributions may vary among tumor subtypes [98,99,100]. (Table 2)

### 4.2. Clinical Validation of microRNAs in Thyroid Cancer

The clinical validation of miRNAs in thyroid cancer has primarily focused on their application as diagnostic biomarkers in molecular assays and, more recently, on their evaluation as therapeutic targets in clinical trials. Growing evidence supports the integration of miRNA profiling into clinical practice, particularly for improving diagnostic accuracy in challenging cases and for guiding personalized treatment strategies.

#### 4.2.1. Validated Diagnostic Panels and Molecular Assays

Recent investigations have emphasized the multicenter validation of miRNA-based molecular tests to enhance the diagnostic assessment of thyroid nodules with indeterminate cytology. In cases where Fine-Needle Aspiration Cytology (FNAC) results are inconclusive, specific miRNA panels have demonstrated substantial diagnostic value by enabling discrimination between benign and malignant lesions. Notably, a panel comprising 19 miRNAs was validated across multiple centers and showed excellent performance in classifying nodules with indeterminate cytology, achieving a sensitivity of 91% and a specificity of 100%. The reliability of individual miRNAs as preoperative diagnostic biomarkers has also been assessed using Ultrasound-Guided Fine-Needle Aspiration Cytology (US-FNAC). Among the most extensively studied miRNAs, miR-146b, miR-221, miR-222, and miR-15a have demonstrated significant discriminative capability, particularly in matched histological samples. MiR-146b exhibited the strongest diagnostic performance, with an Area Under the receiver operating characteristic Curve (AUC) of 0.94, followed by miR-15a (AUC 0.85), miR-221 (AUC 0.79), and miR-222 (AUC 0.76). These findings support the clinical utility of miRNA-based assays as adjunct tools to conventional cytopathological evaluation [100,101].

#### 4.2.2. Validated Circulating miRNAs as Non-Invasive Biomarkers

Circulating miRNAs have attracted increasing attention as minimally invasive biomarkers for long-term surveillance and disease monitoring in thyroid cancer patients. Several studies have validated circulating miRNA signatures detectable in serum or plasma samples. In the postoperative setting, a signature consisting of eight serum miRNAs was identified in patients prior to thyroidectomy. Within this panel, miR-146a-5p and miR-221-3p showed high diagnostic accuracy in distinguishing healthy individuals from patients with persistent or recurrent PTC following treatment, suggesting their potential value in post-surgical follow-up. In MTC, circulating miRNAs have also demonstrated prognostic relevance. Elevated plasma levels of miR-375 have been validated as an independent prognostic marker associated with increased tumor burden and reduced overall survival, underscoring its potential role in risk stratification and outcome prediction [100].

#### 4.2.3. Therapeutic miRNAs Under Clinical Investigation

Although most miRNA-based therapeutic strategies remain at the preclinical stage, several candidates have progressed to clinical evaluation. One of the most advanced examples is *MRX34*, a synthetic miR-34 mimic, which has entered phase I clinical trials for cancer therapy [99]. This milestone highlights the translational potential of miRNA-based therapeutics. In addition, clinical and translational studies have linked miRNA expression profiles to therapeutic response. For instance, miR-375 expression levels have been associated with increased sensitivity to *Vandetanib*, a multi-kinase inhibitor commonly used in the treatment of metastatic MTC. Similarly, miR-199b-5p has been reported to enhance the sensitivity of thyroid cancer cells to *Paclitaxel,* suggesting a possible role for miRNAs in predicting treatment response and overcoming drug resistance [100]. (Table 3)

## 5. Conclusions and Perspectives

**Figure 2:**
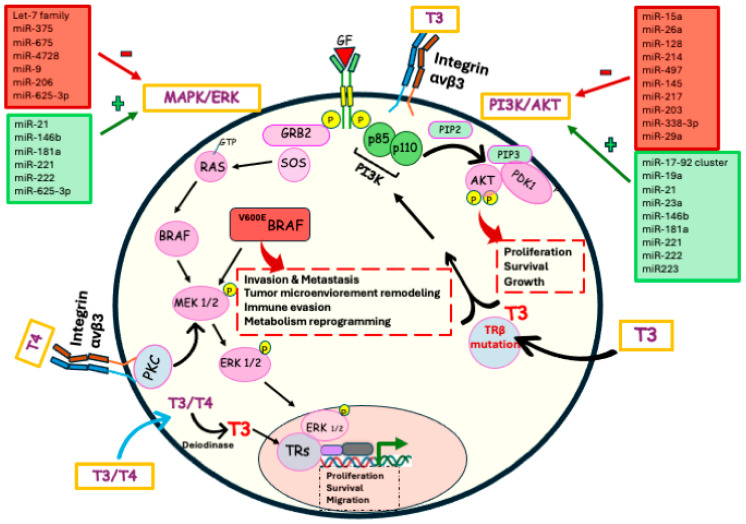
Molecular pathogenesis of Thyroid Cancer: The molecular pathogenesis of thyroid cancer is driven primarily by dysregulation of the MAPK and PI3K signaling pathways. Activation of RTKs by growth factors triggers a cascade that can be altered by multiple oncogenic mutations. Among the most frequent genetic alterations are RAS mutations, which occur across various thyroid cancer subtypes. Another key driver is BRAF, particularly the V600E mutation, which exhibits strong oncogenic potency and leads to constitutive MAPK pathway activation. These pathways are activated when T3 or T4 bind to the membrane receptor integrin αvβ3, initiating downstream activation of MAPK/ERK and PI3K/Akt pathways. As a result, cancer cells enhance proliferation, survival, growth, angiogenesis, invasion, metastasis, immune evasion, and metabolic reprogramming. Furthermore, mutations in TRβ can promote aberrant PI3K/Akt activation, contributing to tumor progression and altered cellular behavior. miRNA dysregulation plays a major role in thyroid carcinogenesis regulation primarily through the regulation of the MAPK/ERK and PI3K/AKT pathways.

In conclusion, thyroid cancer represents a paradigmatic model in which endocrine physiology, oncogenic signaling, tumor microenvironment dynamics, and metabolic reprogramming converge to shape disease initiation and progression. The physiological complexity of TH production and signaling, mediated through tightly regulated genomic and non-genomic mechanisms, underscores the delicate balance required to maintain tissue homeostasis [3,12] (Figure 2).

Disruption of this balance—whether through altered systemic hormone levels, receptor dysfunction, or aberrant intracellular signaling—creates a permissive environment for tumorigenesis. The dual capacity of THs to regulate proliferation, differentiation, metabolism, and inflammation highlights their context-dependent role in cancer biology and reinforces the need to consider endocrine status as an integral component of thyroid tumor pathophysiology [79,86]. In normal physiological conditions, T3 acts as a potent tumor suppressor through nuclear receptors TRs promoting differentiation and exerting anti-proliferative effects [79,86,89]. However, critical conclusions can be drawn regarding how this protective mechanism is subverted in malignancy. The tumor-suppressive influence of TR is frequently lost in thyroid cancer due to epigenetic silencing or somatic mutations, and the mutant receptor constitutively activates the PI3K/Akt pathway, driving uncontrolled proliferation and metastasis [86,89]. Beyond these genomic actions T3 also acts binding to integrin αvβ3 receptor activating the PI3K/AKT cascade [85,86]. Activation of the PI3K/Akt axis has profound effects on cancer cell behavior like promotion of the Warburg effect inducing glycolytic enzymes. Also produce up-regulation of the VEGF-A gene with the resulting induction of angiogenesis [91]. The clinical translation of these molecular findings underscores why elevated T3 levels are a marker of poor outcomes in advanced disease. There is a correlation between higher circulating T3 levels in advanced DTC with aggressiveness due to the loss of genomic growth-inhibitory signals. Because T3 actively supports tumor cell survival through subverted genomic and non-genomic signaling pathways, therapeutic strategies aimed at inducing euthyroid hypothyroxinemia—characterized by reduced circulating T4 and T3 levels while preserving normal systemic metabolism—have emerged as a promising approach to slow disease progression in multiple solid tumors [79,85,89,90]. Preclinical in vitro and xenograft models consistently demonstrate that L-thyroxine (T4), acting via the cell surface receptor on integrin stimulates tumor growth and supports cancer cell defense pathways. Laboratory studies show that T4 at physiological concentrations promotes the proliferation of various thyroid cancer cell lines, whereas T4 analogues like tetrac or its nanoparticulate form, Nano-diamino-tetrac (NDAT) can effectively arrest this growth and restore radiosensitivity. However, a significant discordance is observed when these findings are translated to human populations. A large longitudinal clinical study of patients with intermediate- or high-risk DTC found that elevated Free T4 (FT4) levels were not associated with worse Progression-Free Survival (PFS). This suggests that the potent growth-promoting effects of T4 observed in controlled laboratory environments may not always translate into measurable clinical progression, potentially due to the modest nature of iatrogenic FT4 elevations compared to the higher concentrations sometimes utilized in preclinical models [79,90,103]. It has not even been observed that T4 can induce an activated conformation of integrin αvβ3 and promote radioresistance [90]. The effect of thyroid states varies by cancer type Hypothyroidism, although generally associated with slower progression in several solid tumors like breast and renal cell carcinoma, may paradoxically promote more aggressive behavior in malignancies such as colorectal cancer and hepatocellular carcinoma. In HCC, the overexpression of TSH receptors on cancer cells may allow elevated TSH levels (secondary to hypothyroidism) to directly stimulate tumor progression [79]. While TSH suppression has long been a cornerstone of DTC therapy due to the hormone’s role as a primary growth factor for thyroid follicular cells, recent genetic evidence has introduced an unexpected direction in understanding of its causal role. Traditional clinical views hold that elevated TSH is a risk factor for malignancy and advanced tumor stage. Conversely, Mendelian randomization studies using genetic variants as unbiased proxies suggest that genetically predicted higher TSH levels (indicating lower thyroid function) are actually associated with a reduced risk of thyroid cancer [83,90,94]. This challenges the long-standing view of TSH as a simple causal risk factor and suggests that the majority of TSH-associated genetic variants may act through pathways that independently regulate both thyroid function and growth. Additionally, sex-specific patterns influence cancer incidence at varying TSH levels, with suppressed TSH concentrations linked to higher cancer occurrence in women and elevated TSH representing a more relevant risk factor in men [59,81,82,83]. Standard clinical management for differentiated thyroid cancer commonly includes TSH suppression therapy. This strategy involves the administration of supraphysiological doses of T4 to reduce circulating TSH levels and thereby minimize the proliferative stimulus on residual or recurrent tumor cells. A critical balance exists between TSH-dependence and T4-dependence in thyroid tumors. For most patients, suppressing TSH with T4 successfully arrests tumor growth. However, if a thyroid cancer continues to grow despite full TSH suppression, the tumor may have shifted to becoming T4-dependent, utilizing the integrin αvβ3 pathway to drive its progression [89].

At the molecular level, constitutive activation of the MAPK/ERK and PI3K/AKT pathways represents a central oncogenic driver in thyroid carcinogenesis, linking genetic alterations to the acquisition of key cancer [34,37]. In particular, the ^V600E^BRAF mutation emerges as a master regulator of tumor aggressiveness, promoting epithelial–mesenchymal transition, invasion, metastasis, immune evasion, and metabolic rewiring [39,44,54,65]. Beyond intrinsic tumor cell proliferation, ^V600E^BRAF reshapes the tumor microenvironment by enhancing immune checkpoint expression, recruiting suppressive immune populations, and fostering stromal activation, thereby integrating oncogenic signaling with inflammatory and immunosuppressive networks [53,57,58]. Furthermore, its capacity to drive metabolic reprogramming—including enhanced glycolysis, mitochondrial adaptation, glutamine utilization, and autophagy-mediated therapeutic resistance—highlights the intimate crosstalk between oncogenic pathways and cancer metabolism [37,104]. However, multiple resistance mechanisms have been identified following the use of ^V600E^BRAF inhibitors, challenging the notion of a strictly BRAF-centric paradigm [72,73]. Rather than acting as a solitary driver, ^V600E^BRAF should be considered a key—yet not exclusive—node within a highly adaptive and interconnected signaling network. Resistance to BRAF inhibitors arises from dynamic network-level adaptations—including feedback reactivation of MAPK signaling, activation of alternative pathways such as PI3K/AKT/mTOR, genetic and isoform alterations, phenotypic plasticity, and metabolic rewiring—which collectively diminish tumor dependence on BRAF and limit the durability of therapeutic responses [73,74,75,76]. This perspective underscores the need for combinatorial therapeutic strategies that address feedback reactivation, pathway crosstalk, and tumor plasticity, ultimately aiming to achieve more durable clinical responses.

The evolving histopathological and molecular classification of thyroid neoplasms, particularly following the 5th edition of the World Health Organization (WHO) guidelines, reflects a paradigm shift toward risk-adapted diagnosis and management [17,19]. Although most follicular cell-derived tumors display indolent behavior and favorable outcomes, a subset—including PDTC and ATC—remains highly aggressive and therapeutically challenging. Current treatment strategies, centered on surgery, radioactive iodine, TSH suppression, and tyrosine kinase inhibitors, have significantly improved outcomes in differentiated disease; however, recurrence, radioiodine refractoriness, and treatment-related toxicity remain major clinical limitations [22,23,24]. These challenges underscore the necessity of identifying more precise molecular targets and predictive biomarkers. One of the greatest challenges in the diagnosis of thyroid cancer lies in the pre-surgical assessment of thyroid nodules. Fine-Needle Aspiration Biopsy (FNAB) is a fundamental tool in this context; however, between 10% and 20% of samples are indeterminate and do not allow a definitive diagnosis to be established [95]. This limitation highlights the need to incorporate complementary strategies that optimize diagnostic accuracy before surgery, which could lead to a significant improvement in clinical practice. In this regard, recent research has pointed to the potential of circulating microRNAs—detectable in plasma, serum, urine, or other biological fluids—as a new generation of non-invasive biomarkers for cancer diagnosis. Several studies have demonstrated that distinct histopathological subtypes of thyroid tumors arising from the same cell of origin exhibit specific miRNA expression profiles. Moreover, even within a single tumor type, miRNA signatures may vary according to the presence of oncogenic mutations, underscoring their close association with the molecular landscape of these neoplasms. These findings have important implications for refining the classification of thyroid tumors and improving our understanding of their progression [95,98,99,100]. The integration of these minimally invasive molecular tools into preoperative evaluation algorithms could substantially refine risk stratification and reduce unnecessary surgical interventions. The integration of miRNA-based assays into clinical practice significantly refines the management of indeterminate thyroid nodules, addressing the limitations of traditional FNA. Multicenter validation studies have confirmed that specific miRNA panels, such as a validated 19-miRNA signature, can accurately distinguish between benign and malignant lesions with a sensitivity of 91% and a specificity of 100% [100]. Furthermore, the reliability of molecular testing using US-FNAC has been established for key microRNAs, including miR-146b, miR-15a, miR-221, and miR-222. Among these, miR-146b has demonstrated exceptional diagnostic performance with an AUC of 0.94, followed by miR-15a (0.85), miR-221 (0.79), and miR-222 (0.76) [101]. These tools provide an objective framework to avoid unnecessary diagnostic surgeries for patients with inconclusive cytological results. It has been identified a signature of eight serum miRNAs that allows for effective post-surgical follow-up prior to and after thyroidectomy. Specifically, miR-146a-5p and miR-221-3p have been validated for their high accuracy in discriminating between healthy individuals and those with persistent or recurrent PTC. In the context of MTC, high plasmatic levels of miR-375 serve as a robust, independent prognostic marker associated with increased tumor burden and decreased overall survival. The development of miR-34 mimics, specifically the drug MRX34, is currently being evaluated in phase I clinical trials for cancer treatment. Beyond direct treatment, miRNA expression profiles are critical for predicting and enhancing sensitivity to existing therapies. For instance, levels of miR-375 are linked to improved patient response to vandetanib in metastatic MTC. Additionally, the tumor suppressor miR-199b-5p has been shown to sensitize thyroid cancer cells to paclitaxel, highlighting the potential of miRNAs to overcome chemoresistance and improve therapeutic outcomes. Continued validation in large, multicenter cohorts and standardized assay platforms will be essential for translating these promising tools into routine clinical practice [100].

## Figures and Tables

**Table 1:** WHO 5th Edition Classification of Follicular Cell-Derived Thyroid Neoplasms and Its Clinical Implications. Tumors were categorized using a color-coding system based on the traffic-light model to enhance visual clarity and facilitate rapid risk stratification.

Benign Tumors	Thyroid follicular nodular disease (FND)		
	Follicular adenoma		
	Follicular adenoma with papillary architecture		
	Oncocytic adenoma of the thyroid		
Low-risk Neoplasms	Noninvasive follicular thyroid neoplasm with papillary-like nuclear features (NIFTP)		
	Thyroid tumors of uncertain malignant potential (TTUMP)	Follicular Thyroid tumors of Uncertain Malignant Potential (FT-UMP)	
		Well-Differentiated tumors of Uncertain Malignant Potential (WD-UMP)	
	Hyalinizing Trabecular Tumor (HTT)		
Malignant Neoplasms	LOW-GRADE		
	HIGH-GRADE	Follicular Thyroid Carcinoma (FTC)	
		Invasive Encapsulated Follicular Variant Papillary Carcinoma (IEFV-PTC)	
		Papillary Thyroid Carcinoma (PTC)	
		Oncocytic Carcinoma of the Thyroid (OCA)	
		Follicular-derived thyroid carcinoma	Differentiated High-grade Thyroid Carcinoma (DHGTC)
			Poorly Differentiated Thyroid Carcinoma (PDTC)
		Anaplastic Follicular cell-derived Thyroid Carcinoma (ATA)	

**Table 2:** MicroRNAs involved in thyroid cancer: roles, pathways, molecular targets and effects.

ONCOGENIC miRNAs			
miRNA	Main Pathway	Specific Targets	Biological Effects
miR-17–92 cluster [98,100,101]	PI3K/AKT/mTOR cell cycle	PTEN	Enhances cell proliferation and survival; drives aggressive behavior, particularly in ATC.
miR-21 [100,101]	PI3K/AKT MAPK/ERK NF-κB	PDCD4 PTEN	Promotes cell survival, migration, and invasion; inhibits apoptosis; frequently upregulated in PTC and ATC.
miR-23a [100]	PI3K/AKT	PTEN	Promotes cell proliferation and invasion
miR-146b [101]	MAPK/ERK PI3K/AKT TGF-β	PTEN SMAD4	Promotes proliferation, migration, invasion, and EMT; strongly associated with BRAF V600E mutation and aggressive PTC features.
miR-155 [98,99,101]	β-catenin TGF-β	APC TP53INP1	Enhances tumor growth and invasion; activates β-catenin/c-MYC signaling; associated with extrathyroidal extension in ATC.
miR-181a [101]	PI3K/AKT MAPK/ERK	PTEN RASSF1A	Promotes proliferation, migration, and invasion; associated with tumor size, lymph node metastasis, and increased recurrence risk in PTC.
miR-221 miR-222 [98,99,101]	MAPK/ERK PI3K/AKT cell cycle	CDKN1B KIT	Induces cell-cycle progression; increases proliferation, invasion, recurrence risk, and lymph node metastasis.
miR-223 [101]	PI3K/AKT NF-κB	FBXW7 EPB41L3	Promotes cell proliferation and invasion; correlates with tumor size, lymph node metastasis, and recurrence risk in PTC.
miR-625-3p [98]	WNT/JNK	AEG-1	enhances tumor cell migration, invasion, and proliferation, promotes drug tolerance, and suppresses apoptosis
TUMOR SUPPRESSOR miRNAs			
miRNA	Main Pathway	Specific Targets	Biological Effects
let-7 family [98,99]	MAPK/ERK	RAS HMGA2	Suppresses cell proliferation, migration, and invasion; promotes differentiation; loss contributes to malignant transformation.
miR-9	MAPK/ERK	BRAF	
miR-15a [98,101]	PI3K/AKT apoptosis	BCL-2	Promotes apoptosis and limits proliferation; controversial role in thyroid cancer depending on subtype and context.
miR-26a [98]	PI3K/AKT epigenetic regulation	MRTFA RAP1B	Inhibits tumor growth and proliferation; particularly relevant in ATC.
miR-128 [98,100,101]	PI3K/AKT Sphingolipid metabolism	SPHK1	Inhibits tumor growth and reduces tumor burden in thyroid cancer models.
miR-137 [98,99]	CXCL12/CXCR4 axis	CXCL12	Inhibits PTC cell proliferation, migration, and invasion.
miR-153-3p [98,99]	RET signaling	RET	Suppresses RET-dependent oncogenic signaling; inhibits MTC tumor growth and invasion; synergistic with cabozantinib.
miR-200 family [98,100,102]	EMT-related pathways	ZEB1 ZEB2	Inhibits epithelial–mesenchymal transition; loss promotes invasion and metastasis, particularly in ATC.
miR-206 [98]	Cell migration cytoskeleton regulation	MRTFA RAP1B	Suppresses migration and invasion of PTC and ATC cells; restoration reduces aggressive behavior.
miR-214 [98,100,102]	Proteasome pathway	PSMD10	Inhibits proliferation and epithelial–mesenchymal transition in thyroid cancer cells.
miR-375 [98,99,100]	RET ERBB signaling	ERBB2	Suppresses migration and invasion in MTC; involved in neuroendocrine differentiation.
miR-675	MAPK/ERK	MAPK1	
miR-497 miR-29a miR-145 miR-217 miR-203 miR-338-3p [98]	PI3K/AKT	AKT3	Inhibit tumor cell proliferation, migration, and invasion, reduce metastatic potential, and suppress tumor growth both in vitro and in vivo.
miR-4728 [100]	MAPK/ERK	SOS1	Inhibits cell proliferation

**Table 3:** Clinical validation of microRNAs in Thyroid Cancer.

Clinical Application	miRNA(s)	Sample Type	Clinical Use	Performance Findings
Diagnostic classification of indeterminate nodules [100,101]	19-miRNA panel	FNAC samples from indeterminate thyroid nodules	Differentiation between benign and malignant lesions	91% sensitivity and 100% specificity
Preoperative diagnostic assessment [101]	miR-146b miR-221 miR-222 miR-15a	USFNAC and histological samples	Preoperative discrimination between benign and malignant thyroid lesions	High diagnostic accuracy in histology
Post-surgical Monitoring [100]	miR-146a-5p miR-221-3p	Serum	Detection of persistent or recurrent PTC after thyroidectomy	High accuracy in distinguishing healthy subjects from patients with persistent or recurrent disease
Prognostic Stratification [100]	miR-375	Plasma	Prognostic biomarker in MTC	Elevated levels associated with higher tumor burden and worse overall survival; independent prognostic marker
Therapeutic Development [99]	miR-34 mimic, MRX34	Systemic administration	miRNA-based cancer therapy	Evaluated in phase I clinical trials
Prediction of drug Response [100]	miR-375	Tumor tissue	Prediction of response to targeted therapy in MTC	Higher miR-375 levels associated with increased sensitivity to vandetanib
Chemotherapy Sensitization [100]	miR-199b-5p	Tumor cells (experimental/ clinical data)	Enhancement of chemotherapy efficacy	Increases sensitivity of thyroid cancer cells to paclitaxel

## Data Availability

The original contributions presented in this study are included in the article. Further inquiries can be directed to the corresponding author.
